# Botanic Origin of Propolis Extract Powder Drives Contrasted Impact on Diabesity in High-Fat-Fed Mice

**DOI:** 10.3390/antiox10030411

**Published:** 2021-03-09

**Authors:** Nicolas Cardinault, Franck Tourniaire, Julien Astier, Charlene Couturier, Lauriane Bonnet, Eva Seipelt, Esma Karkeni, Claire Letullier, Naima Dlalah, Stephane Georgé, Lourdes Mounien, Jean-Francois Landrier

**Affiliations:** 1 Pollenergie, La Grabère, 47450 Saint Hilaire de Lusignan, France; nicolas.cardinault@gmail.com (N.C.); claire.letullier@pollenergie.fr (C.L.); 2 Aix Marseille University, C2VN, INRAE, INSERM, 13000 Marseille, France; feukauff@yahoo.fr (F.T.); julien.astier@univ-amu.fr (J.A.); charlene.couturier@univ-amu.fr (C.C.); bonnet.lauriane19@gmail.com (L.B.); seipelt.eva@gmail.com (E.S.); esma1304@hotmail.fr (E.K.); lourdes.mounien@univ-amu.fr (L.M.); 3 CriBioM, Criblage Biologique Marseille, Campus Santé Timone, 13000 Marseille, France; 4 Biochemistry Department, Centre Technique de Conservation des Produits Agricoles (CTCPA), site Agroparc, 84911 Avignon, France; Naima.Dlalah@inrae.fr (N.D.); sgeorge@ctcpa.org (S.G.)

**Keywords:** adipose tissue, glucose tolerance, obesity, propolis, preventive nutrition

## Abstract

Propolis extracts are considered as nutraceutical products with potentialities towards obesity and comorbidities management. Nevertheless, propolis extracts composition is highly variable and depends on the botanic origin of plants used by the bees to produce propolis. This study aims to evaluate the differential effect of poplar propolis extract powder (PPEP), *Baccharis* propolis extract powder (BPEP), and/ or *Dalbergia* propolis extract powder (DPEP) on obesity and glucose homeostasis in high-fat-fed mice. PPEP supplementation reduced high-fat (HF)-mediated body weight gain, adiposity index, and improved glucose homeostasis in male C57Bl/6J mice that were submitted to a high-fat diet for 12 weeks, whereas BPEP, DPEP, or a mix of the three PEPs did not modify those parameters. Adipose tissue (AT) gene expression profiling highlighted an induction of mRNA related to lipid catabolism and an inhibition of mRNA coding for inflammatory markers. Several Nrf2 target genes, coding for antioxidant enzymes, were induced in AT under PPEP effect, but not by other PEP. Interestingly, representative PPEP polyphenols mediated the induction of Nrf2 target genes cell-autonomously in adipocytes, suggesting that this induction may be related to the specific polyphenol content of PPEP. Whereas PPEP supplementation has demonstrated a clear potential to blunt the onset of obesity and associated comorbidities, other PEPs (from *Baccharis* and *Dalbergia*) were inefficient to support their role in preventive nutrition.

## 1. Introduction

Propolis is a resinous substance collected by bees from buds, trees, or shrubs in the plant ecosystem close to the hive [1]. Propolis has been used as a folk medicine in Europe, Asia, and South America, [2,3]. The various biological and pharmacological effects reported for propolis activity are related to the phenolic compounds, which can widely vary in quality and in quantity, depending on the plant source and the site of collection [1]. Three types of propolis have been subjected to in-depth studies in terms of identification, characterization, and biological activities [1,4,5], i.e., the brown propolis from *Populus* sp. (poplar) produced in temperate zones, characterized by the presence of the polyphenolic taxonomic markers such as pinocembrin, chrysin, and galangin, and esters of substituted cinnamic acids, especially phenylethyl caffeate (CAPE) [6]. The green propolis from *Baccharis dracunculifolia* from the Minas Gerais region in Brazil is characterized by the presence of artepellin C, p-coumaric, and drupanin as taxonomic markers [7,8], and the red propolis from *Dalbergia ecastophyllum* from the region of Algolas in Brazil is identified by the specific presence of isoflavans, isoflavons, or pterocarpans class represented by vestitol, formononetin, and medicarpin [5,9].

Interestingly, preclinical studies performed in animal models of obesity and/or diabetes have reported beneficial phenotypical impact of propolis (mainly as ethanolic extracts) supplementation on these noncommunicable diseases [10,11,12,13,14,15]. Such preventive approach, based on nutraceutical use of propolis, would be relevant to tackle obesity and associated disorders, including type 2 diabetes (T2DM). Indeed, obesity is considered as a major public health problem throughout the world, and a major risk factor of T2DM, which represents 90–95% of diabetes mellitus cases worldwide [16]. T2DM is a systemic disease strongly linked to obesity-mediated, low-grade inflammation. Such process, which corresponds to an increased production of mediators of the immune response [17], including cytokines [17], chemokines [18], and microRNA [19,20,21] by adipose tissue (AT), and a reduction of adiponectin plasma level [22], interferes with insulin signaling in insulin-sensitive tissues (liver, skeletal muscle, and AT), and contributes to the etiology of T2DM [23].

All the studies depicting potential interest of propolis extracts supplementation to limit obesity and comorbidities have used propolis from different origins, suggesting that various types of propolis are able to mediate similar effects. Nevertheless, propolis extracts used in these studies result from different preparative methods, have been administrated at different doses (with a content of active ingredients not known), using different ways of administration and vehicles to deliver extracts [11,12,14,24,25,26,27]. Therefore, it is difficult to properly interpret those results and to compare efficiency of the different types of propolis, since no study has been designed to compare the effect of propolis extracts from different botanical origins administered at the same dose in active principles, on the same set of experiments. Thus, the aim of the present study is to evaluate the ability of different botanical types of propolis extract powder (PEP) from poplar, *Baccharis dracunculifolia,* or *Dalbergia escastaphyllum*, to limit body weight gain and glucose homeostasis disruption in diet-induced obese mice.

## 2. Materials and Methods

### 2.1. Propolis Powder Preparation

All propolis samples were gathered according to special specifications to minimize their contamination. Pollenergie selected these supplies from beekeepers from the southern regions of France for poplar propolis, from the Minas Gerais region in Brazil for *Baccharis* propolis, and from the Alagoas region in Brazil for *Dalbergia* propolis. These three propolis samples were initially analyzed by GC-MS in Vassya Bankova Laboratory (Laboratory of chemistry of natural compounds, Sofia, Bulgaria) in order to confirm the botanical origin of each sample by comparing the polyphenolic imprint to the taxonomic markers of the source plant [28]. The identification of the main molecules, listed in Table 1, in different samples confirmed their botanical origin. Samples were kept frozen and stored at −20 °C. The propolis powders were prepared from ethanolic extracts. Ethanolic extract of propolis was prepared using the method previously described [29]. Briefly, the raw propolis was cut into small pieces, incubated in 75% ethanol for 8 days, filtered twice, and then evaporated at 50 °C using a rotary evaporator (Evaporator E100). The dry residue of each botanical propolis type was then reduced to powder by grinding by adding food excipients to give the propolis extract powder (PEP) used in the present study.

### 2.2. Total and Individual Polyphenols Quantification

Total polyphenol content was measured according to the method described by Popova et al. [30]. Briefly, 1 g of each PEP was dissolved in 30 mL of ethanol (70%) in a 50 mL Falcon tube and heated for 2 h at 70 °C. After centrifugation, the ethanolic phase was recovered and the extraction was repeated twice. All supernatants were combined and adjusted to 100 mL with 70% ethanol in a volumetric flask. PEP solutions were finally diluted 1 to 50 times in methanol/water (1:1). Standard solutions were prepared using the following protocol. A blank tube was prepared with 100 µl of 70% ethanol and 100 µl of standard mix of diluted concentrations, and 1.5 mL of distilled water was added with 0.4 mL of Folin–Ciocalteu reagent. The tubes were vortexed, and 0.6 ml of Na_2_CO_3_ 20% and 2.4 mL of distilled water were added and mixed. The tubes were incubated at 50 °C for 15 min. After cooling at room temperature, absorbance was measured at 760 nm. Samples were diluted 1/10 with 70% ethanol before analysis. Of diluted extracts of propolis, 100 µL was placed into tubes, and the procedure was the same for the standard. Results were expressed as milligrams of pinocembrin/galangin (2:1 (*w/w*)) equivalent per 100 g of powder of poplar propolis and as gallic acid equivalent for *Baccharis* and *Dalbergia* propolis [28]. The analyses were performed in triplicate. Polyphenol profile was determined by HPLC as previously reported by Gardana et al. [31] for poplar propolis extract powder (PPEP) and *Baccharis* propolis extract powder (BPEP). Separation was achieved by HPLC using a Symmetry (250 × 4.6 mm, C18) column (Waters, Saint Quentin en Yvelines, France). Samples were eluted with formic acid 0.1% (solvent A) and acetonitrile (solvent B) at a flow rate of 1.2 mL/min. A stepwise gradient was employed, starting at 80% A and 20% B and changing to 30% B in 10 min, to 40% B in 40 min, to 50% B in 50 min, to 100% B in 55 min during 5 min; the gradient returned to initial condition at 62 min for the last 8 min. DAD data were acquired in the 200–450 nm range. Specifically, isoflavones, which are specific markers of *Dalbergia* propolis extract powder (DPEP) and found only in the propolis of this botanic origin, were determined by HPLC as previously reported [9]. The DPEP solution was acidified with trifluoroacetic acid, and the separation was achieved by HPLC using a Kinetex C18 100 × 4.6 mm, 2.6 µm column (Phenomenex, France). Samples were eluted with formic acid 1% (solvent A) and acetonitrile with 1% formic acid (solvent B) at a flow rate of 1.2 mL/min. A stepwise gradient was employed, starting at 80% A and 20% B and changing to 30% B in 10 min, to 40% B in 40 min, to 60% B in 60 min, and to 98% B in 80 min during 10 min. DAD data were acquired in the 200–482 nm range. Polyphenolic compounds and isoflavones were identified by comparing their retention time and UV characteristics to standards. The composition in terms of individual polyphenol for each PEP solution is reported on Table 1.

### 2.3. Animal Experiments

The protocol received the agreement of the local ethics committee and the French Minister of Research and Education (ethical approval number n°01549.04). Six-week-old male C57BL/6J mice were obtained from Janvier Labs (Le Genest Saint Isle, France) and fed ad libitum with control food (chow diet A04 from Safe-diets) during a 1-week acclimation period, with full access to drinking water. The animals were maintained at 22 °C under a 12 h light, 12 h dark cycle at 20% humidity level. Mice (10 per group) were randomly assigned into one of the six experimental groups depending on the diet, that is, control (chow diet A04 from Safe diets), high fat (HF: 45% energy from lipids, Test Diet ref. 58V8), HF supplemented with PPEP (4.5 mg of total polyphenols/mouse/day), HF supplemented with BPEP (4.5 mg of total polyphenols/mouse/day), HF supplemented with DPEP (4.5 mg of total polyphenols/mouse/day), or HF supplemented with mix (mix of the 3 PEPs, 1.5 mg of PPEP, BPEP, and DPEP, the sum corresponding to 4.5 mg of total polyphenols/mouse/day). PEPs were mixed in the food in a quantity depending of their respective total polyphenol concentration to deliver a similar amount of total polyphenol per day for 12 weeks, that is, 4.5 mg of total polyphenol/mouse/day. Body weight evolution was followed once a week, and food intake was measured every two weeks. Twelve weeks after the beginning of the protocol, mice were fasted overnight. Blood sampling was performed by cardiac puncture under general anesthesia. Plasma was prepared by centrifugation at 3000 rpm for 15 min at 4 °C and was stored at −80 °C. Mice were euthanized by cervical dislocation under general anesthesia, and various tissues (liver and various white AT deposits) were collected, weighted, and stored at −80 °C.

### 2.4. Oral Glucose Tolerance Test

Two weeks before the end of the protocol, mice were subjected to glucose tolerance tests. To this aim, overnight-fasted (16 h) mice were gavaged with glucose (2 g/kg). Whole blood was drawn from tail tip for glucose measurements (Accu-Check glucometer, Roche Diagnostic, Meylan, France).

### 2.5. Biochemical Analyses

All parameters have been quantified as previously reported [32,33]. Briefly, glucose concentration in plasma was evaluated using glucose RTU (bioMerieux, Craponne, France). Triglycerides and free fatty acids (FFAs) were measured by colorimetric methods (RANDOX, Crumlin, Co. Antrim, United Kingdom). Insulin was measured using an enzyme-linked immuno-sorbent assay ELISA (ALPCO Diagnostics, New Hampshire, United States). Leptin and adiponectin were quantified by ELISA (R&D Systems quantikine ELISA). β-hydroxybutyrate concentration was measured using a colorimetric test according to the manufacturer’s procedure (BEN srl, Milano, Italy). The HOMA-IR index was calculated according to the following formula: fasting insulin (µU/L) × fasting glucose (nmol/L)/22.5.

### 2.6. Cell Culture

3T3-L1 preadipocytes (ATCC, Manassas, VA) were seeded in 3.5 cm diameter dishes at a density of 15 × 10^4^ cells/well. Cells were grown in DMEM supplemented with 10% FBS at 37 °C in a 5% CO_2_ humidified atmosphere, as previously reported [34,35,36]. To induce differentiation, two-day postconfluent 3T3-L1 preadipocytes (Day 0) were stimulated for 48 h with 0.5 mM isobutylmethylxanthine, 0.25 μM dexamethasone, and 1 μg/mL insulin in DMEM supplemented with 10% FBS. The cultures were then treated with DMEM supplemented with 10% FBS and 1 μg/mL insulin. To examine the impact of purified polyphenols on gene expression, 3T3-L1 adipocytes were incubated with various concentrations of polyphenols (chrysin, galangin, pinocembrin, and caffeic acid phenethyl ester (CAPE)) dissolved in ethanol at 0.1% for 24 h. Control received the vehicle (ethanol) alone. Concentrations used were calculated to correspond to the concentrations of polyphenols in a solution of 30 µg/mL of PEP, on the basis of their respective quantities determined in PEP. All treatments were performed on Day 8. The data are the mean of three independent experiments, each performed in triplicate.

### 2.7. RNA Extraction and Real-Time PCR

Total RNA was extracted from epididymal AT or from cells using TRIzol reagent (Thermo, Courtaboeuf, France), as previously described [37]. One microgram of total RNA was used to synthetize cDNAs using random primers and Moloney murine leukemia virus reverse transcriptase (Thermo, Courtaboeuf, France). Real-time quantitative PCR analyses were performed using the AriaMX real-time PCR system (Agilent Technologies France, Les Ullis) as previously described [38]. For each condition, expression was quantified in duplicate, and 18S rRNA was used as the endogenous control in the comparative cycle threshold (CT) method [39]. Primers used are listed in Table 2.

### 2.8. Statistical Analysis

The data are communicated as the mean ± SEM. Significant differences between the control and treated groups were determined using ANOVA, followed by the Fisher’s (least significant difference (LSD) post hoc test using GraphPad Prism. *p* < 0.05 was considered statistically significant.

## 3. Results

### 3.1. Polyphenols Quantification in the Different Propolis Extract Powders

As a first experiment, the total quantity of polyphenols was determined (Table 1) to provide similar amounts of total polyphenols to mice in the different groups. Then, the individual polyphenol profile and content of the three different PEPs were determined (Table 1).

### 3.2. Propolis Extract Powder Supplementation Differentially Protects Against Diet-Induced Obesity

**Figure 1:**
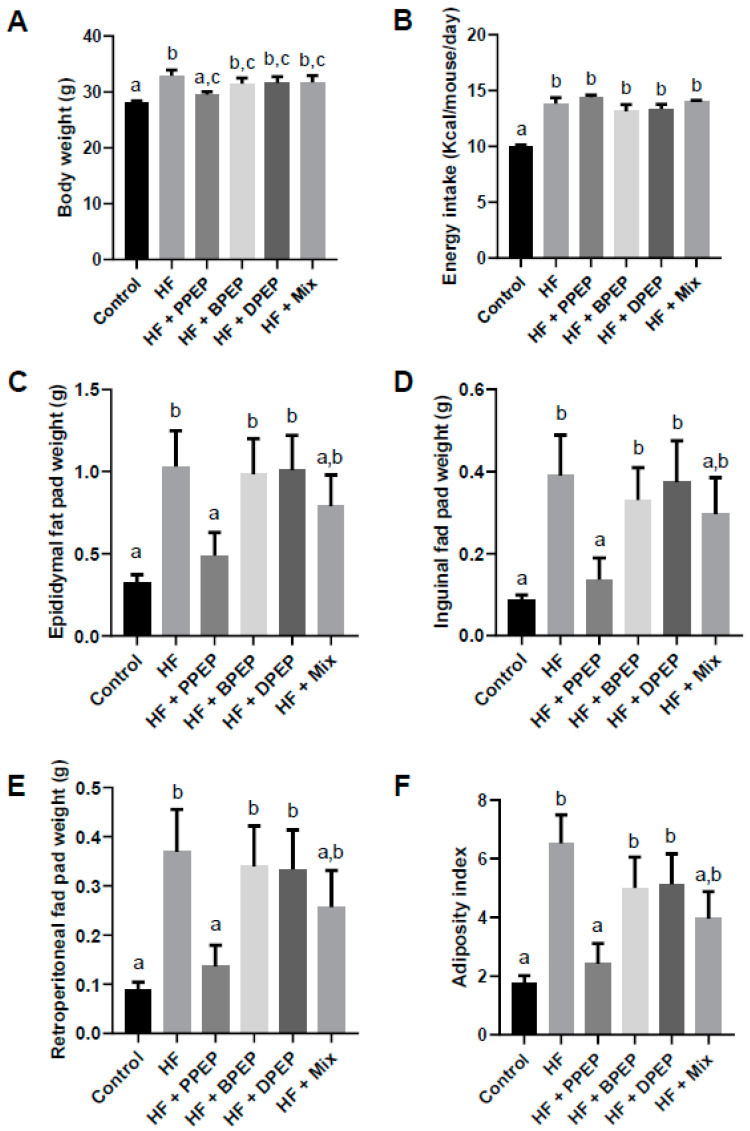
Propolis extract powder differentially limits weight gain associated with diet-induced obesity. (**A**) Mice were weighed at the end of the protocol (*n* = 10 per group). (**B**) Food intake was estimated to determine energy intake every two weeks for a period of 10 weeks. (**C**) Absolute epididymal fat pad weight (grams). (**D**) Absolute inguinal fat pad weight (grams). (**E**) Absolute retroperitoneal fat pad weight (grams). (**F**) Adiposity index was determined by dividing the sum of adipose tissues weight by the body weight of animal. Values are presented as means ± SEM. Bars not sharing the same letter were significantly different in Fisher’s LSD post hoc test *p* < 0.05. PPEP: poplar propolis extract powder; BPEP: *Baccharis* propolis extract powder; DPEP: *Dalbergia* propolis extract powder; Mix: mixture of the 3 propolis extract powders.

The effect of the supplementations with the three PEPs and a mix of the three PEPs (at the same quantity of total polyphenols, i.e., 4.5 mg of total polyphenols/mouse/day) were evaluated in C57BL/6J mice submitted to a HF diet for 12 weeks. Only PPEP supplementation significantly limited weight gain compared with the HF diet (Figure 1A). Other PEPs and the mix did not modify body weight compared with the HF group. The energy intake of the HF group was higher than that of the control group, but was not impacted by the various PEP supplementations, compared to the HF group (Figure 1B). Body weight gain limitation in PPEP-supplemented mice was associated with an absolute fat mass limitation of all different fat pads measured (epididymal, retroperitoneal, or inguinal; Figure 1C–E). Consequently, the adiposity index (sum of epididymal, inguinal, and retroperitoneal AT mass relative to total body mass) of PPEP-supplemented mice was significantly reduced, compared with HF-fed mice (Figure 1F). BPEP, DPEP, and the mix did not modify the absolute fat pad masses or the adiposity index compared with the HF group. Only the mix tended to reduce all these parameters, but did not reach statistical significance. Plasma biochemical parameters were quantified (Table 3). Triglyceride levels were only modestly decreased by BPEP. No modulation of nonesterified fatty acids and alanine transaminase (ALAT) levels was observed in HF or PEP-supplemented groups. Adiponectin plasma level was not modified by PPEP supplementation compared to HF group but was significantly reduced compared with the control group. Supplementation with BPEP, DPEP, and the mix increased adiponectin levels compared with the HF group. Leptin plasma level increased in the HF group compared with the control group and reduced in the PPEP-supplemented group, but was not modified by other PEPs. β-hydroxybutyrate level was increased in the HF group compared with the control group and was reduced by all PEPs except DPEP.

### 3.3. Poplar Propolis Extract Powder Supplementation Improves Glucose Homeostasis

**Figure 2:**
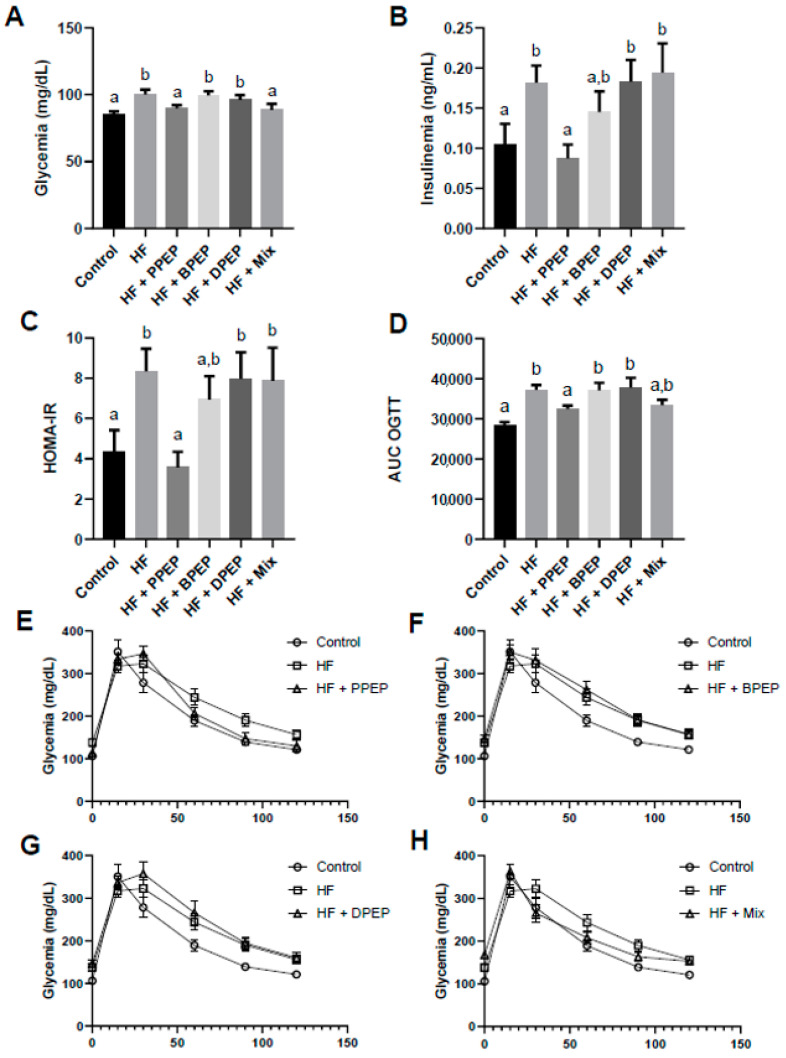
Propolis extract powder differentially improves glucose homeostasis. (**A**) Glycemia of mice was determined in fasted state (*n* = 10 mice per group). (**B**) Insulinemia was determined in fasted state. (**C**) HOMA-IR mean values were calculating using fasted glycemia and insulinemia. (**D**) Area under the curve for the glycemic response during oral glucose tolerance test (OGTT). (**E**–**H**) Glycemic evolution following the OGTT (*n* = 10 mice per group). Values are presented as means ± SEM. Bars not sharing the same letter were significantly different in Fisher’s LSD post hoc test *p* < 0.05. PPEP: poplar propolis extract powder; BPEP: *Baccharis* propolis extract powder; DPEP: *Dalbergia* propolis extract powder; Mix: mixture of the 3 propolis extract powder.

The effect of PEP supplementations on glucose homeostasis was evaluated in fasted animals. Glycemia was increased by HF diet and normalized compared with the control group in PPEP- and mix-supplemented mice (Figure 2A). Plasma insulin was higher in the HF group, but only PPEP supplementation normalized this parameter compared to the control group (Figure 2B). HOMA-IR index was increased by HF diet and corrected by PPEP supplementation. Other PEP supplementations did not significantly improve HOMA-IR compared to the HF group (Figure 2C). Additionally, the impact of PEP supplementations was also evaluated through oral glucose tolerance test (OGTT). PPEP and mix supplementations improved glucose tolerance in OGTT (Figure 2E,H), as highlighted by area under the curve (AUC) of the glycemic response (Figure 2D), which was smaller compared with the HF-fed mice.

### 3.4. Poplar Propolis Extract Powder Supplementation Modifies Gene Expression in Adipose Tissue

**Figure 3:**
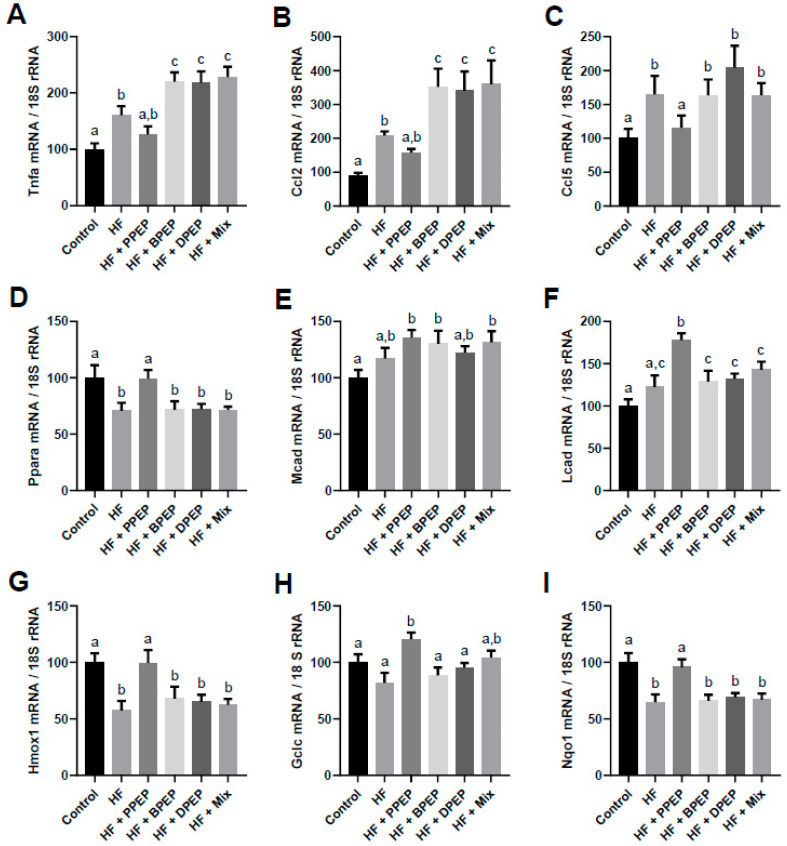
Propolis extract powder differentially impacts gene expression in adipose tissue. (**A**–**C**) Relative expression of genes related to inflammatory markers measured by qPCR in epididymal adipose tissue measured through qPCR and expressed relative to 18S ribosomal RNA. (**D**–**F**) Relative expression of genes related to lipid metabolism measured through qPCR in epididymal adipose tissue and expressed relative to 18S ribosomal RNA. (**G**–**I**) Relative expression of Nrf2 target genes coding for antioxidant enzymes measured through qPCR in epididymal adipose tissue and expressed relative to 18S ribosomal RNA. *n* = 10 mice per each group, values are presented as means ± SEM. Bars not sharing the same letter were significantly different in Fisher’s LSD post hoc test *p* < 0.05. PPEP: poplar propolis extract powder; BPEP: *Baccharis* propolis extract powder; DPEP: *Dalbergia* propolis extract powder; Mix: mixture of the 3 propolis extract powder.

The effect of the various PEP supplementations was evaluated on AT inflammation. The expression of several mRNAs coding for markers of inflammation such as Tnf-α and chemokines, including chemokine C-C motif ligand 5 (Ccl5) and Ccl2, was quantified. As expected, the HF diet increased these mRNA levels compared with the control group. Interestingly, PPEP supplementation decreased the mRNA level of these inflammatory markers (Figure 3A−C), whereas other PEPs and the mix further increased the expression of those mRNAs (Figure 3A,B) or did not modify the expression level compared to the HF group.

The expression of genes encoding transcription factors involved in energy metabolism and fatty acid oxidation was studied in order to explain the differential AT mass accumulation. mRNA levels coding for the peroxisome proliferator-activated receptor α (Ppar-α) were downregulated in HF-fed mice compared with the control group. Interestingly, PPEP supplementation restored the mRNA levels of Ppar-α, whereas BPEP, DPEP, and the mix did not induce any modification compared to the HF group (Figure 3D). The medium-chain acyl-CoA dehydrogenase (Mcad) mRNA was induced by all PEPs, except DPEP, compared to control group, but was not statistical different from that of the HF group (Figure 3E). The long-chain acyl-CoA dehydrogenase (Lcad) mRNA levels were also induced by the PPEP, compared to HF-fed mice, but the other supplementations did not modulate its expression compared to the HF group (Figure 3F).

Since nuclear factor erythroid 2–related factor 2 (Nrf2) signaling is suspected to mediate the effects of PEP, the activation of this pathway was investigated. Several target genes of Nrf2, including heme oxygenase 1 (Hmox1), glutamate-cysteine ligase catalytic subunit (Gclc), and NADPH: quinone oxidoreductase 1 (Nqo1), were evaluated. Hmox1 and Nqo1 genes were downregulated by the HF diet compared to the control diet (Figure 3G,I), and Gclc was only slightly and not significantly reduced by the HF diet (Figure 3H). PPEP supplementation restored the downregulated mRNA levels of Hmox1 and Nqo1, but the other PEPs or the mix were inefficient (Figure 3G,I). Regarding Gclc mRNA, only PPEP significantly induced its expression compared to HF or control groups (Figure 3H).

### 3.5. Polyphenols Present in Poplar Propolis Extract Powder Modify Gene expression in Adipocytes

**Figure 4:**
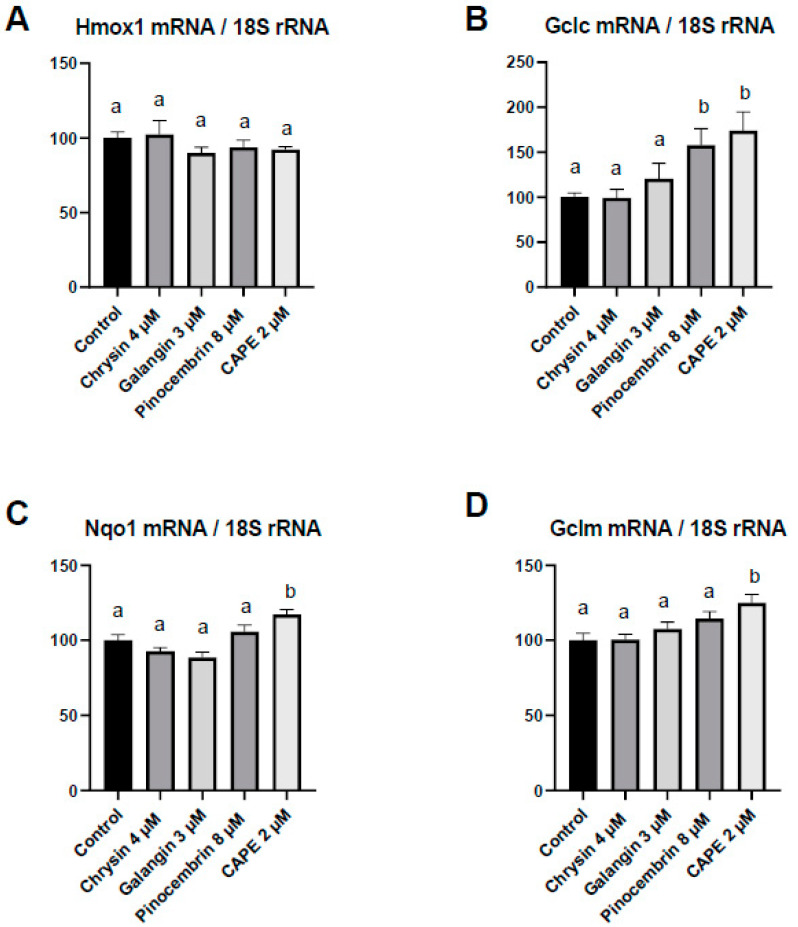
Polyphenols differentially impact gene expression in adipocytes. (**A**–**D**) Relative expression of Nrf2 target genes (Hmox1, Gclc, Nqo1, and Gclm) coding for antioxidant enzymes measured through qPCR and expressed relative to 18S ribosomal RNA. 3T3-L1 adipocytes were incubated with chrysin (4 µM), galangin (3 µM), pinocembrin (8 µM), or CAPE (2 µM) for 24 h. Values are presented as means ± SEM. Bars not sharing the same letter were significantly different in Fisher’s LSD post hoc test *p* < 0.05.

To go further in the identification of the bioactive molecules present in PPEP that may be responsible for the Nrf2 signaling activation, the ability of the main PPEP polyphenols to modulate Nrf2 target genes was evaluated in adipocytes. If the expression of Hmox1 mRNA level was not modified by polyphenol incubation in adipocytes, Gclc gene was induced by both pinocembrin and CAPE, whereas Nqo1 and Gclm genes were induced only by CAPE (Figure 4).

In the present study, we compared the efficiency of three different PEPs and a mix of these PEPs to blunt obesity and associated disruption of glucose homeostasis mediated by an HF diet in mice.

The PEPs used in the present study have been extracted with the same methodology from propolis of three different botanic origins and are known to display distinct phytochemical compositions, in agreement with quantifications of polyphenols presently reported. Indeed, the PPEP was prepared from propolis collected from *Populus sp*., in temperate zones, and is characterized by flavonoids without B-ring substituents (including pinocembrin, pinobanksin, galangine, and chrysin), and phenylpropanoid acids and their esters (caffeic acid phenethyl ester: CAPE). The BPEP was prepared from green propolis collected from *Baccharis dracunculifolia* in the Minas Gerais region in Brazil. This propolis is characterized by prenylated phenylpropanoids (artepellin C) and caffeoylquinic acids. Finally, the DPEP was prepared from red propolis whose source is *Dalbergia ecastophyllum* from the region of Algolas in Brazil. This extract is mainly characterized by the high presence of isoflavonoids (formononetin) [5]. Thus, the polyphenolic fraction of these different extracts is very different from one botanical source to another and thus constitutes a kind of specific fingerprint for each propolis type. It also suggests that the biological effects mediated by the different extracts may differ depending on their specific polyphenols.

In agreement, we demonstrated that the PPEP supplementation limited weight gain, mainly via a limitation of fat mass accretion as reflected by the reduced adiposity index in mice HF-diet-fed mice, similarly to previous reports [27,40,41]. Such effect was associated to a decrease of leptin plasma levels, which correlated to the reduction in mass of the different fat pads [42]. Surprisingly, BPEP and DPEP did not induce such body weight limitation and limitation of fat mass accumulation, whereas the mix of the three PEPs resulted in a clear tendency to reduce fat mass accumulation and adiposity index. This last effect could be due to the fact that one-third of the mix was provided by PPEP. Nevertheless, the fact that both BPEP and DPEP were inefficient is surprising since many studies already reported a beneficial impact of Brazilian propolis regarding metabolic disorders. Indeed, several studies using Brazilian propolis supplementation in HF-fed rats [10] or mice [11,12,13] reported a limitation of fat mass. If the botanical origin of the Brazilian propolis was not mentioned in some studies [10,12,13], Sakai et al. indicated that *Baccharis dracunculifolia* was the main botanical source used in their experiments [11]. It is noteworthy that the quantity of artepellin C (major phenolic acid of Brazilian propolis) found in the propolis used by Sakai et al. was four times higher than in our BPEP, and could explain in part the conflicting results. Nevertheless, the origin of such discrepancies is presently not clear, as several parameters could be involved, such as the duration of the treatment, the doses used, the galenic to deliver the molecules (propolis or extracts), the administration way, and finally, the animal model used.

Interestingly, the role of CAPE to mediate beneficial effects of PPEP could be evoked, since the anti-obesity effect of this compound has already been reported [43], and it is noteworthy that this molecule is present in PPEP, but not in BPEP, in our extracts. In addition, this molecule was able to induce cell-autonomously the upregulation of several Nrf2 target genes in adipocyte cultures, suggesting that this bioactive compound may mediate the transactivation of the Nrf2 signaling pathway, possibly involved in the anti-obesity effects of the PPEP [40].

Since we [40] and others [41] have reported that the weight gain limitation under PPEP was correlated to an induction of several genes related to fatty acid oxidation/browning in white AT, we explored, in the present study, the expression of genes linked to lipid metabolism. As anticipated, PPEP supplementation, but not other PEPs or the mix, induced the expression of Ppar-α, Mcad, and Lcad, suggesting that PPEP activated a fatty acid oxidative program, known to be associated with enhanced energy expenditure [44]. Such mechanism is highly suspected to explain, at least in part, the anti-obesity effect for natural bioactive compounds such as all-trans retinoic acid [36], vitamin D [45,46], or lycopene [32].

Besides the effect on lipid metabolism, we also investigated the impact of supplementations on inflammatory marker expression, since it is well established that AT accumulation leads to the establishment of a low-grade inflammatory status in AT [17]. PPEP supplementation reduced the tunoral necrosis factor α (Tnf-α), C-C Motif Chemokine Ligand 2 (Ccl2), and 5 (Ccl5) mRNA levels, whereas BPEP and DPEP slightly induced Tnf-α and Ccl2 mRNA levels and did not modify the expression of Ccl5. These data suggest that some phenolic compounds present in BPEP and DPEP may have a direct pro-inflammatory effect in AT since those extracts did not increase the fat mass, resulting in an indirect pro-inflammatory effect. This point remains unclear and will require further experiments.

The AT inflammatory status plays a major role in the disruption of glucose homeostasis in the context of obesity. Thus, we evaluated the impact of PEP supplementation on fasted glycemia, insulinemia, HOMA-IR, and glycemia evolution during an oral glucose tolerance test. All these parameters were improved in HF-fed mice supplemented with PPEP, which is in agreement with the previous data. Indeed, propolis extracts have been depicted to restore glucose tolerance and insulin sensitivity/secretion in several type 2 diabetes animal models [13,14,15,26,27,47]. Nevertheless, homeostasis of only some of glucose parameters was improved by the other supplements: fasted glycemia and the area under the curve of the OGTT was improved by the mix, and plasma insulin tended to be reduced by the BPEP. If the slight effect of the mix could be easily explained by the presence of PPEP in this mix that drove beneficial effect, the impact of BPEP on plasma insulin is less clear, but it is suggested that BPEP could partly restore insulin sensitivity [27]. The mechanism behind the effect is presently not known and will deserve further experiments.

Several molecular mechanisms have been proposed to explain the biological effect of propolis, including the Nrf2 signaling pathway. Indeed, we recently demonstrated that PPEP transactivated Nrf2 signaling in AT. Such activation could be involved in the reported PPEP effect on obesity and associated disorders [40], since several Nrf2 agonists, including CDDO-lm [48], oltipraz [49], glycyrrhizin [50], or glucoraphanin [51], were able to blunt the HF-mediated obesity and insulin resistance in rodents. In addition, several bioactive molecules found in propolis are able to activate the Nrf2 signaling pathway [52], including CAPE [53], as suggested by cell culture experiments presented here. Nevertheless, the mechanisms by which Nrf2 activators restore energy homeostasis in a diet-induced obesity mice model remains elusive, and several hypotheses have been emitted, including induction of lipid oxidation and limitation of oxidative stress and inflammation, which display an important role in energy homeostasis and insulin sensitivity disruption [54,55].

Nrf2 belongs to the cap’n’collar-type transcription factor family (for review [56,57,58]). Bioactive compound inducers of Nrf2 will upregulate several genes, including Nqo1, Gclc, and Hmox-1 coding for proteins of the antioxidant system and detoxification system. In line with the putative involvement of this signaling pathway, we reported in the present study that several Nrf2 target genes (Hmox1, Gclc, and Nqo1) were upregulated by PPEP and CAPE, but not by other PEPs, supporting the role of the Nrf2 activation in the metabolic PPEP-mediated effects, via a CAPE-dependent mechanism.

## 4. Conclusions

To conclude, our data strongly support the specific role of PPEP as an interesting nutraceutical approach to blunt HF-mediated obesity and glucose homeostasis disruption. Note that the quantity of total polyphenols provided to mice corresponds to a dose of 12 mg of total polyphenol/kilogram of body weight in human, taking into account the conversion factor for dose translation between mice and human [59]. Such dosage corresponds to a physiological dose of polyphenol (around 840 mg for a 70 kg adult subject), compared to the mean polyphenol intake in human (1 g/day; [60]). We can easily hypothesize that this effect depends on the specific phenolic compound profile of PPEP, especially the presence of CAPE. Nevertheless, we have to keep in mind that such profile is subjected to variations from batches to batches and from years to years. Therefore, a process of standardization would be required to provide PEP with a consistent bioactive compound profile, to warrant a consistent effect on diet-induced obesity limitation.

## Figures and Tables

**Table 1:** Polyphenols content of the different propolis powder extracts.

Polyphenol (mg/100 g)	Poplar Propolis Extract	*Baccharis* Propolis Extract	*Dalbergia* Propolis Extract
Caffeic acid	373	87	Nm
Coumaric acid	1654	728	Nm
Ferulic acid	381	28	Nm
Apigenin	137	Nd	Nm
CAPE	246	Nd	Nm
Quercetin	94	213	Nm
Kaempferol	368	288	Nm
Galangin	1598	Nd	Nm
Cinnamic acid	789	Nd	Nm
Pinocembrin	2349	73	Nm
Chrysin	1850	Nd	Nm
Artepillin C	Nd	1503	Nm
Formononetin	Nm	Nm	1010
Biochanin A	Nm	Nm	167
Liquiritigenin	Nm	Nm	1075
Vestitol	Nm	Nm	9741
Medicarpin	Nm	Nm	4317
Total polyphenols (g/100 g extract powder)	41.1	20	29.5

Nd: non detected; Nm: not measured; CAPE: phenylethyl caffeate.

**Table 2:** Primers sequences.

Gene Name	Accesion Number	Forward Primer	Reverse Primer
*Tnfα*	NM_013693.3	CATCTTCTCAAAATTCGAGTGACAA	TGGGAGTAGACAAGGTACAACCC
*Pparα*	NM_011144.6	CTGAGACCCTCGGGGAAC	AAACGTCAGTTCACAGGGAAG
*Mcad*	NM_007382.5	TGTCGAACACAACACTCGAAA	CTGCTGTTCCGTCAACTCAA
*Lcad*	NM_007381.4	TGGGGACTTGCTCTCAACA	GGCCTGTGCAATTGGAGTA
*Ccl2*	NM_011333.3	CATCCACGTGTTGGCTCA	GATCATCTTGCTGGTGAATGAGT
*Ccl5*	NM_013653.3	TGCAGAGGACTCTGAGACAGC	GAGTGGTGTCCGAGCCATA
*Hmox1*	NM_010442.2	AGGCTAAGACCGCCTTCCT	TGTGTTCCTCTGTCAGCATCA
*Gclc*	NM_010295.2	AGATGATAGAACACGGGAGGAG	TGATCCTAAAGCGATTGTTCTTC
*Gclm*	NM_008129.4	TGACTCACAATGACCCGAAA	TCAATGTCAGGGATGCTTTCT
*Nqo1*	NM_008706.5	AGCGTTCGGTATTACGATCC	AGTACAATCAGGGCTCTTCTCG
*18S*	NR_003278.3	CGCCGCTAGAGGTGAAATTCT	CATTCTTGGCAAATGCTTTCG

**Table 3:** Biochemical parameters of mice.

	Control	HF	HF + PPEP	HF + BPEP	HF + DPEP	HF + Mix
**TG (mmol/L)**	1.195 ± 0.11 a	1.092 ± 0.12 a,c	0.93 ± 0.03 a	0.822 ± 0.13 b,c	0.92 ± 0.05 a	1.15 ± 0.08 a
**NEFA (mmol/L)**	1.075 ± 0.12 a	1.041 ± 0.09 a	0.94 ± 0.07 a	1.005 ± 0.007 a	1.052 ± 0.115 a	1.071 ± 0.08 a
**Β-OH butyrate (mmol/L)**	2.319 ± 0.15 a	4.051 ± 0.35 b	2.44 ± 0.26 a	3.13 ± 0.48 a,b	3.26 ± 0.41 b	2.89 ± 0.30 a
**ALAT (UI)**	2479 ± 313 a	2225 ± 400 a	2592 ± 529 a	2659 ± 64 3 a	1980 ± 263 a	2175 ± 732 a
**Adiponectin (ng/mL)**	6836 ± 370 a	5072 ± 776 b,c	4712 ± 287 b	6369 ± 609 a	6860 ± 576 a	6401 ± 508 a,c
**Leptin (pg/mL)**	1660 ± 658 a	12029 ± 2961 b,c	2900 ± 2038 a	8323 ± 2246 a,c	11196 ± 2995 b	9774 ± 3368 b

TG: triglycerides; NEFA: nonesterified fatty acids; B-OH butyrate: β-hydroxy butyrate; ALAT: alanine transaminase. PPEP: poplar propolis extract powder; BPEP: *Baccharis* propolis extract powder; DPEP: *Dalbergia* propolis extract powder; Mix: mixture of the 3 propolis extract powders. Values are reported as means ± SEM. Bars not sharing the same letter were significantly different in ANOVA followed by a Fisher’s least significant difference (LSD) post hoc test at *p* < 0.05.

## Data Availability

The data presented in this study are available on request from the corresponding author. The data are not publicly available due to privacy.
